# Supplementary material for: Signatures of time interval reproduction in the human electroencephalogram (EEG)
Source: Imaging Neurosci (Camb). 2024 Aug 29;2:imag-2-00279. doi: 10.1162/imag_a_00279 (PMC12290544; doi:10.1162/imag_a_00279)
Supplement: Supplementary Material [file imag_a_00279-supp.pdf]

Supplemental Materials

S1: Strength of sample interval effect varied across participants; previous trial sample interval did not predict reproduction time.

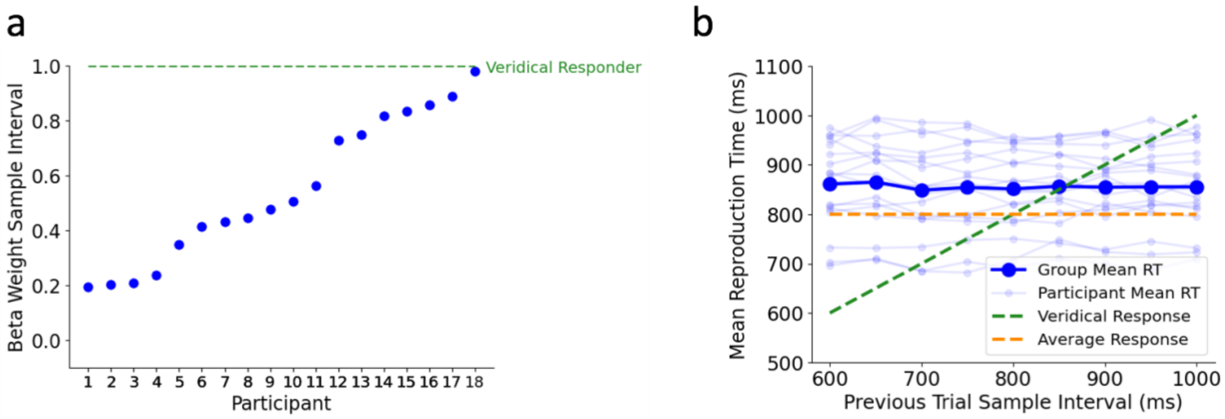

Figure S1: a) The degree to which Reproduction Time was influenced by Sample Interval varied substantially across participants. Each point represents the beta coefficient for Sample Interval in a single participant linear regression predicting Reproduction Time. b) Group mean Reproduction Time (dark blue) and individual participant mean Reproduction Time (light blue) as a function of previous trial sample interval duration.

S2: EEG Signatures at Sample Interval Offset

- No effect of Sample Interval on CNV amplitude ($x^2(1) = 0.12$, $p = 0.73$, $b = 0.005$) or slope ($x^2(1) = 0.21$, $p = 0.65$, $b = 0.005$).
- No effect of RT Bin on CNV amplitude ($x^2(1) = 2.71$, $p = 0.1$, $b = -0.006$) or CNV slope ($x^2(1) = 0.005$, $p = 0.94$, $b = 6.28e-4$).

- No effect of Sample Interval on Mu/Beta amplitude ($x^2(1) = 0.006$, $p = 0.94$, $b = -8.5e-4$) or slope ($x^2(1) = 0.77$, $p = 0.38$, $b = -0.01$).
- No effect of RT Bin on Mu/Beta amplitude ($x^2(1) = 8.23e-7$, $p = 0.97$, $b = -1.47e-4$) or slope ($x^2(1) = 0.034$, $p = 0.85$, $b = -0.002$)

- No effect of Sample Interval on CPP amplitude ($x^2(1) = 0.19$, $p = 0.66$, $b = 0.005$) or slope ($x^2(1) = 0.11$, $p = 0.74$, $b = -0.003$).
- No effect of RT Bin on CPP amplitude ($x^2(1) = 0.44$, $p = 0.51$, $b = -0.002$) or CPP slope ($x^2(1) = 5.86e-4$, $p = 0.98$, $b = -7.46e-4$).

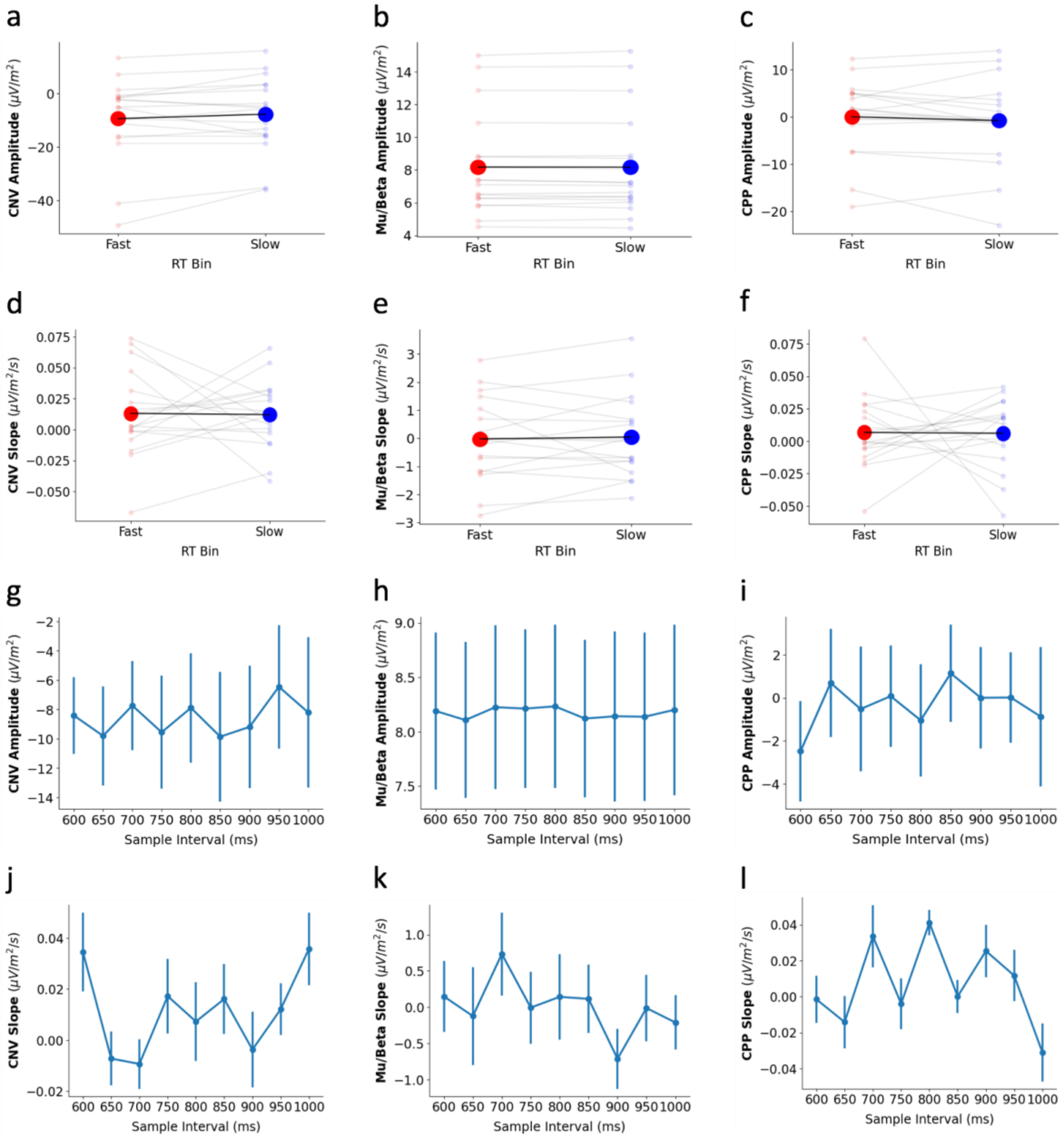

Figure S2: Group mean (black lines and large markers) and participant mean (grey lines and small markers) amplitude (first row) and build-up rate (second row) as a function of RT Bin at sample interval offset, as well as amplitude (third row) and build-up rate (fourth row) as a function of Sample Interval at sample interval offset. First column is CNV (a, d, g, j), second column is Mu/Beta (b, e, h, k) and third column is CPP (c, f, i, l).

S3: Waveforms Separated by Sample Interval

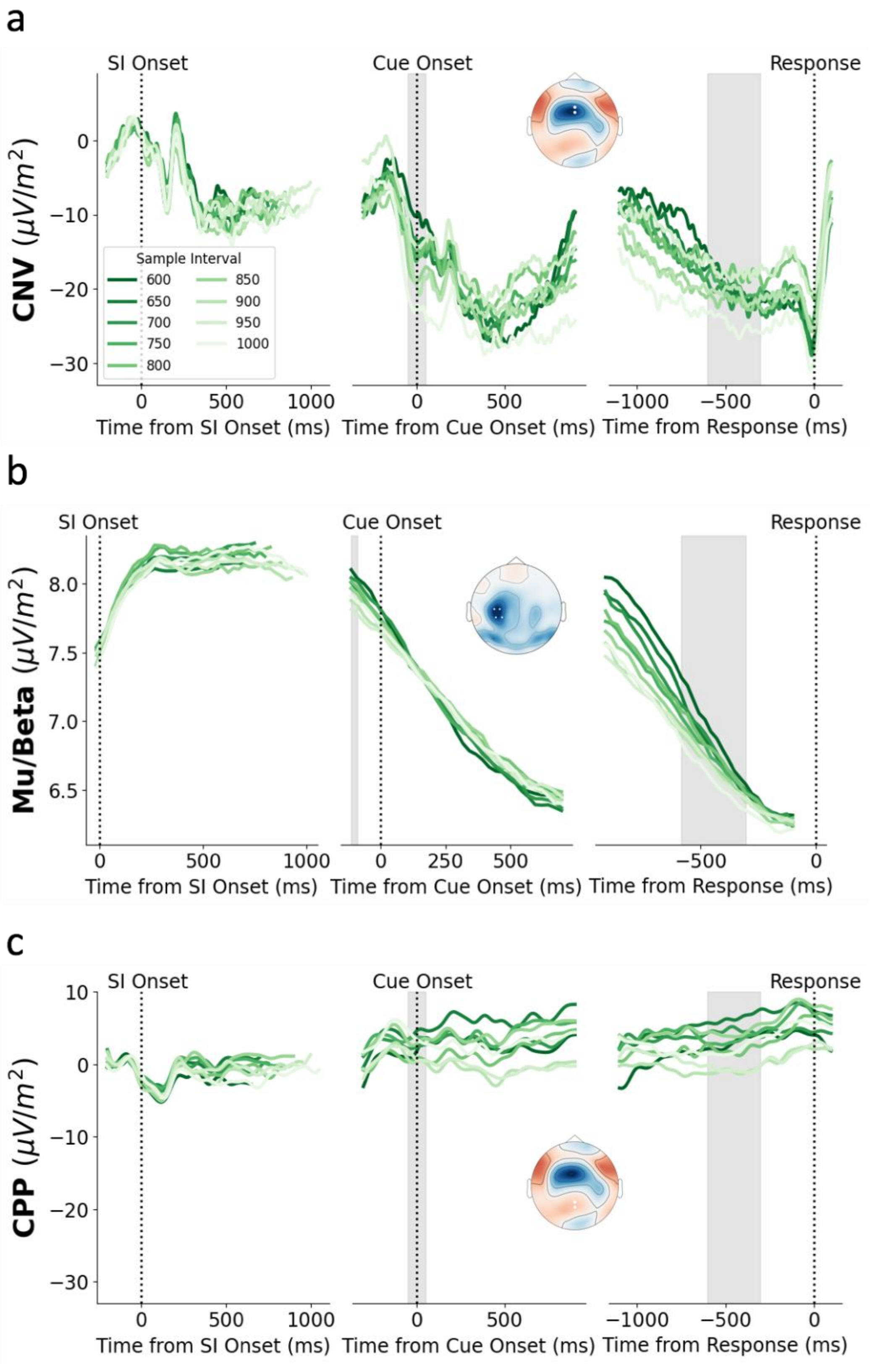

Figure S3: Average waveforms and topographies of the CNV (a), Mu/Beta (b) and CPP (c) across the three epochs as a function of Sample Interval.

S4: Cue Onset and Pre-Response Measure Lineplots

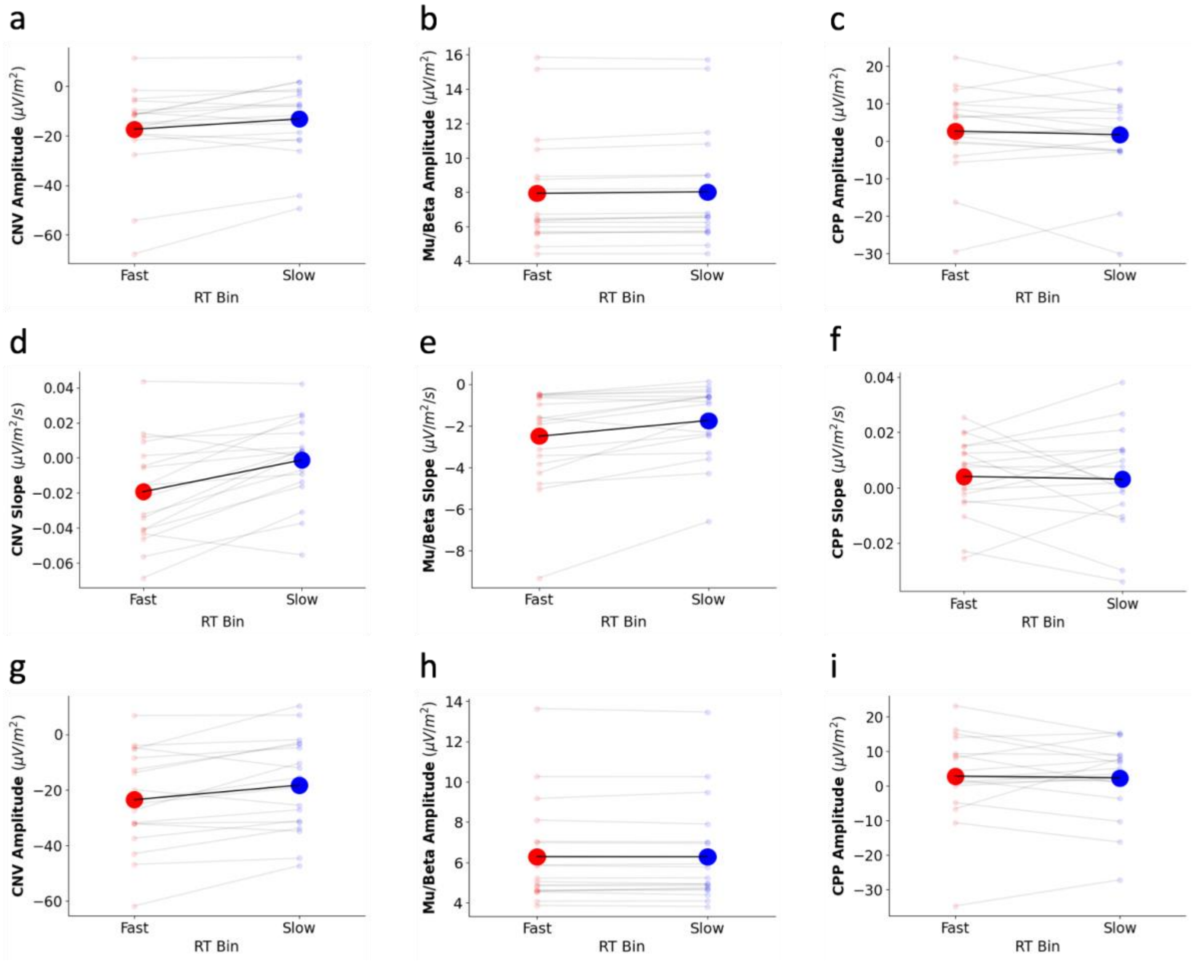

Figure S4: Group mean (black lines and large markers) and participant mean (grey lines and small markers) cue onset amplitude (first row), pre-response build-up rate (second row) and pre-response amplitude as a function of RT Bin. First column is CNV (a, d, g), second column is Mu/Beta (b, e, h) and third column is CPP (c, f, i).

S5: RT Bin ANOVAs

Encode Offset

- No effect of RT bin on CNV amplitude ($F(1, 17) = 1.80$, $p = 0.20$, $\eta^2_p = 0.1$)
- No effect of RT bin on CNV slope ($F(1, 17) = 3.7e^{-5}$, $p = 0.92$, $\eta^2_p = 5.48e^{-4}$)
- No effect of RT bin on Mu/Beta amplitude ($F(1, 17) = 4.28e^{-5}$, $p = 0.80$, $\eta^2_p = 0.004$)
- No effect of RT bin on Mu/Beta slope ($F(1, 17) = 1.66e^{-4}$, $p = 0.74$, $\eta^2_p = 0.006$)
- No effect of RT bin on CPP amplitude ($F(1, 17) = 0.97$, $p = 0.34$, $\eta^2_p = 0.05$)
- No effect of RT bin on CPP slope ($F(1, 17) = 1.8^{-5}$, $p = 0.95$, $\eta^2_p = 2.38e^{-4}$)

Cue Onset

- Significant effect of RT bin on CNV amplitude ($F(1, 17) = 6.43$, $p = 0.02$, $\eta^2_p = 0.27$)
- Significant effect of RT bin on Mu/Beta amplitude ($F(1, 17) = 7.86$, $p = 0.01$, $\eta^2_p = 0.32$)
- No effect of RT bin on CPP amplitude ($F(1, 17) = 0.46$, $p = 0.51$, $\eta^2_p = 0.03$)

Reproduction

- Significant effect of RT bin on CNV amplitude ($F(1, 17) = 10.50$, $p = 0.005$, $\eta^2_p = 0.38$)
- Significant effect of RT bin on CNV slope ($F(1, 17) = 19.09$, $p < 0.001$, $\eta^2_p = 0.53$)
- No effect of RT bin on Mu/Beta amplitude ($F(1, 17) = 3.85e^{-5}$, $p = 0.83$, $\eta^2_p = 0.003$)
- Significant effect of RT bin on Mu/Beta slope ($F(1, 17) = 12.74$, $p = 0.002$, $\eta^2_p = 0.43$)
- No effect of RT bin on CPP amplitude ($F(1, 17) = 3.69e^{-5}$, $p = 0.74$, $\eta^2_p = 8.42e^{-4}$)
- No effect of RT bin on CPP slope ($F(1, 17) = 4.34e^{-5}$, $p = 0.78$, $\eta^2_p = 0.005$)

S6: Separating Motor Preparation Signature into Alpha Mu (8-13Hz) and Beta (15-30Hz) bands

It is common to combine contralateral pre-motor alpha mu and beta as they tend to exhibit highly similar dynamics (DeLange et al., 2013; Murphy et al., 2016; Steinemann et al., 2018; Kelly et al., 2021). However, previous work has shown a relationship between the phase-amplitude coupling of occipital alpha and fronto-central beta, suggesting that the two frequencies bands play distinct roles in timing (Grabot et al., 2019; Kononowicz et al., 2019).

Therefore, we plotted activity in the mu (8-13Hz) and beta (15-30Hz) bands separately and re-ran the analyses including Signal Type (Mu vs Beta) as a fixed effect in the LMM. In the Cue-Onset and Reproduction epochs, mu and beta follow a similar trajectory. We replicated the main effects of RT Bin, Sample Interval, and Gap on pre-response slope (RT Bin: $x^2(1) = 26.78$, $p < 0.001$, $b = -0.03$; SI: $x^2(1) = 14.40$, $p < 0.001$, $b = 0.02$; Gap: $x^2(1) = 5.00$, $p = 0.03$, $b = -0.01$) and cue-onset amplitude (RT Bin: $x^2(1) = 4.9$, $p = 0.03$, $b = -0.01$; SI: $x^2(1) = 4.12$, $p = 0.04$, $b = - 0.01$; Gap: $x^2(1) = 9.1$, $p = 0.003$, $b = - 0.01$), while observing no significant interactions between the main effects of RT Bin, Gap, or Sample Interval and Signal Type (all $p > 0.05$).

The biggest difference between mu and beta occurred during sample interval presentation. While beta amplitude initially increased for 250ms following Sample Interval Onset before beginning to decrease, mu amplitude increased throughout sample interval presentation. The LMM replicated the results reported in the text that there was no significant effect of Sample Interval on the amplitude or slope of the signal at Sample Interval Offset (Amplitude: $x^2(1) = 1.9$, $p = 0.16$, $b = 0.01$; Slope: $x^2(1) = 1.43$, $p = 0.23$, $b = -0.01$), but there was a significant interaction between Sample Interval and Signal Type (Amplitude: $x^2(1) = 41.45$, $p < 0.001$, $b = -0.03$; Slope: $x^2(1) = 4.303$, $p = 0.04$, $b = 0.01$). There was no significant effect of RT Bin on signal slope or amplitude at Sample Interval Offset (Amplitude: $x^2(1) = 0.24$, $p = 0.62$, $b = -0.002$; Slope: $x^2(1) = 0.04$, $p = 0.85$, $b = 0.001$).

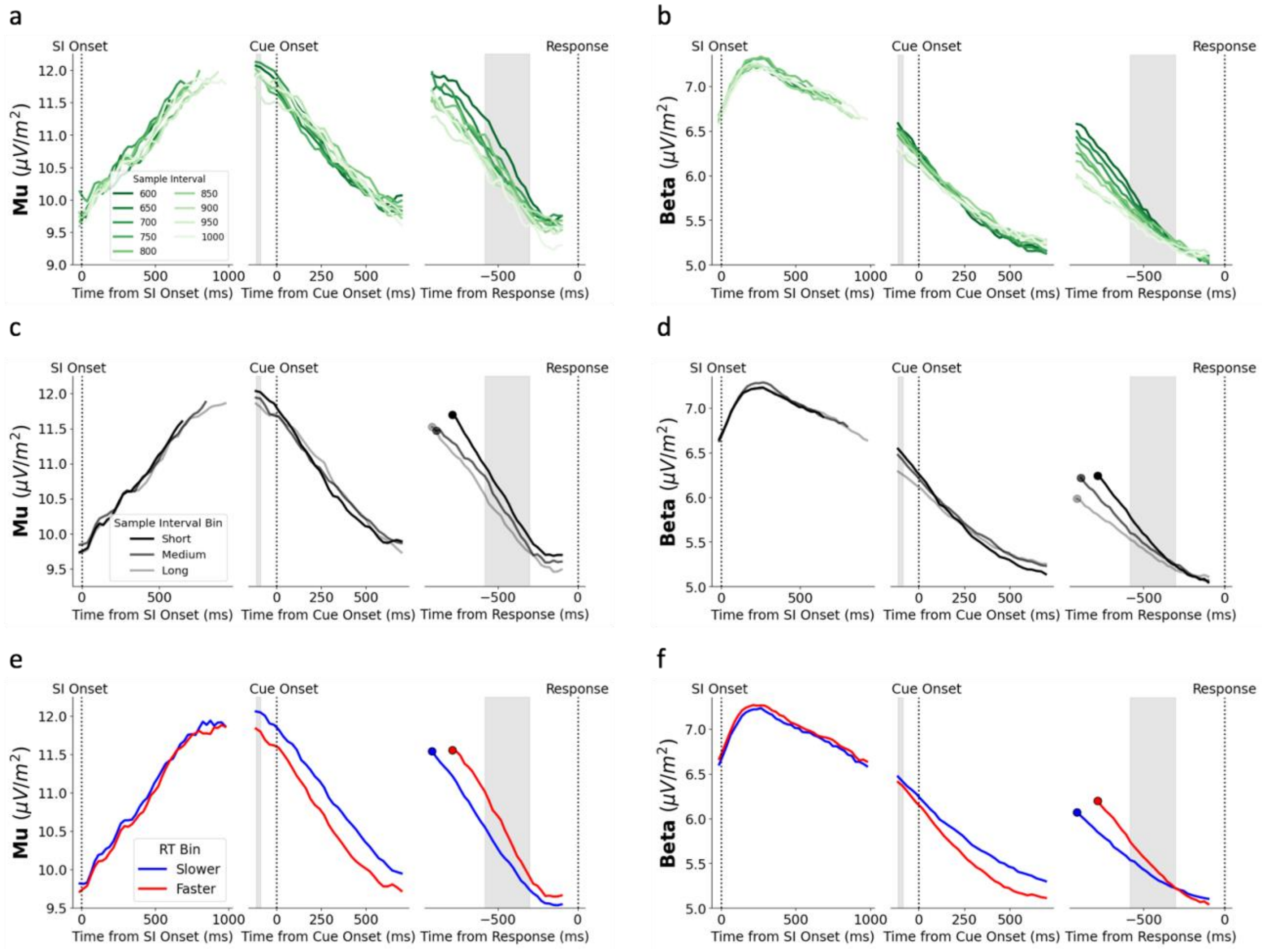

Figure S6: Average waveforms for Mu (first column) and Beta (second column) across the three epochs as a function of Sample Interval (first row: a and c), Sample Interval Bin (second row: c and d) and RT Bin (third row: e and f).

S7: P2 at Sample Interval Offset

Previous research has indicated that the amplitude of offset potentials following the end of the sample interval is related to sample interval duration (Konowicz & Van Rijn, 2014; Damsma et al., 2021; Bueno & Cravo; 2021; Kruijne et al., 2021; Ofir & Landau, 2022). Therefore we examined the amplitude of the P2 at sample interval offset. This was also important to check as an effect in this time period could have overlapped and contaminated the effects we examined at cue onset. Following the approaches in previous papers, we measured the offset amplitudes at the same electrodes as the CNV (FCz), and baselined the data from -50ms to 50ms from sample interval offset (Damsma et al., 2021; Kononowicz & Van Rijn, 2014). The waveform plots below highlight a very small positive going potential that peaks circa 150 ms after offset and which may correspond to the P2 offset response. We measured the mean amplitude of the P2 in a window of 100ms to 200ms and assessed the effect of Sample Interval on its amplitude using a LMM with Sample Interval as a fixed effect, including random intercepts and random slopes across participants as random effects. This model indicated that there was no significant effect of Sample Interval on P2 amplitude at Sample Interval Offset (x2(1) = 0.6, p = 0.43, b = 0.01). We also found no effect of RT Bin on P2 amplitude at sample interval offset (x2(1) = 0.01, p = 0.94, b = -0.0001). We also repeated these analyses for mu/beta, and the findings are consistent with the response preparation dynamics we report at cue onset. Mu/beta desynchronisation builds steadily throughout the gap between sample interval offset and cue onset and there is no sign of an evoked response following sample interval offset (Fig S7 f, g, h). In the same time window as the above analyses (100ms to 200ms following sample interval offset) mu/beta desynchronisation was greater for longer sample intervals ($x^2(1) = 5.01$, $p = 0.03$, $b = -0.02$). There was no significant difference in the level of mu/beta desynchronisation between RT Bins in the 100ms to 200ms post-sample interval offset ($x^2(1) = 0.61$, $p = 0.44$, $b = -0.004$), but a significant difference emerged using a later time window between 250ms and 350ms, with a greater level of mu/beta desynchronisation for faster responses ($x^2(1) = 5.34$, $p = 0.02$, $b = -0.01$).

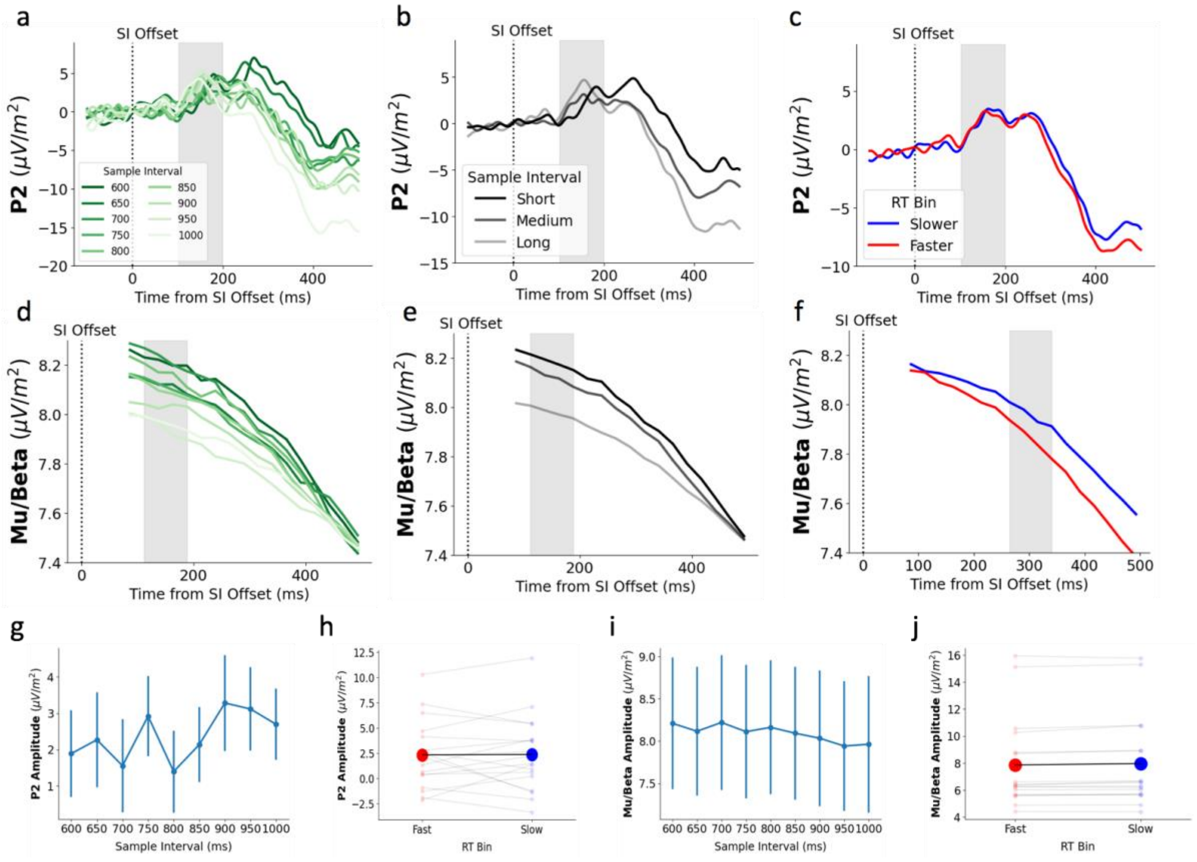

Figure S7: P2 waveforms plotted at sample interval offset as a function of Sample Interval (a), Sample Interval Bin (b) and RT Bin (c). Mu/beta waveforms plotted at sample interval offset as a function of Sample Interval (d), Sample Interval Bin (e) and RT Bin (f). P2 amplitude (100ms to 200ms post-sample interval offset) as plotted as a function of Sample Interval (g) and RT Bin (h). Mu/Beta amplitude as plotted as a function of Sample Interval (i) and RT Bin (j).
